# Structural and Functional Characteristics of Color Vision Changes in Choroideremia

**DOI:** 10.3389/fnins.2021.729807

**Published:** 2021-10-07

**Authors:** Jasleen K. Jolly, Matthew P. Simunovic, Adam M. Dubis, Amandeep S. Josan, Anthony G. Robson, Marco P. Bellini, Edward Bloch, Odysseas Georgiadis, Lyndon da Cruz, Holly Bridge, Robert E. MacLaren

**Affiliations:** ^1^Nuffield Laboratory of Ophthalmology, Nuffield Department of Clinical Neurosciences, University of Oxford, Oxford, United Kingdom; ^2^Oxford Eye Hospital and NIHR Oxford Biomedical Research Centre, Oxford University Hospitals NHS Foundation Trust, Oxford, United Kingdom; ^3^Oxford Centre for Functional MRI of the Brain (FMRIB), Wellcome Centre for Integrative Neuroimaging, University of Oxford, Oxford, United Kingdom; ^4^Save Sight Institute, Discipline of Ophthalmology, University of Sydney, Sydney, NSW, Australia; ^5^Retinal Unit Sydney Eye Hospital, Sydney, NSW, Australia; ^6^NIHR Biomedical Resource Centre at Moorfields Eye Hospital and UCL Institute of Ophthalmology, London, United Kingdom; ^7^Electrophysiology Department, Moorfields Eye Hospital, London, United Kingdom; ^8^University College London Institute of Ophthalmology, London, United Kingdom

**Keywords:** color vision, choroideremia, cones, retinal degeneration, ellipsoid zone, pattern electroretinogram, Cambridge color test

## Abstract

Color vision is considered a marker of cone function and its assessment in patients with retinal pathology is complementary to the assessments of spatial vision [best-corrected visual acuity (BCVA)] and contrast detection (perimetry). Rod-cone and chorioretinal dystrophies—such as choroideremia—typically cause alterations to color vision, making its assessment a potential outcome measure in clinical trials. However, clinical evaluation of color vision may be compromised by pathological changes to spatial vision and the visual field. The low vision Cambridge Color Test (lvCCT) was developed specifically to address these latter issues. We used the trivector version of the lvCCT to quantify color discrimination in a cohort of 53 patients with choroideremia. This test enables rapid and precise characterization of color discrimination along protan, deutan, and tritan axes more reliably than the historically preferred test for clinical trials, namely the Farnsworth Munsell 100 Hue test. The lvCCT demonstrates that color vision defects—particularly along the tritan axis—are seen early in choroideremia, and that this occurs independent of changes in visual acuity, pattern electroretinography and ellipsoid zone area on optical coherence tomography (OCT). We argue that the selective loss of tritan color discrimination can be explained by our current understanding of the machinery of color vision and the pathophysiology of choroideremia.

## Introduction

The primary retinal neurons for conscious vision are duplex, consisting of two broad types: the rods and the cones (intrinsically photosensitive retinal ganglion cells—ipRGCs—which primarily drive circadian function and the pupillary light reflex are not considered further in this paper). In the healthy retina, rods confer scotopic (nocturnal) function; whilst cones are primarily used for photopic (daylight level) function. Although the cones are present throughout the retina, their high density in the fovea is responsible for high spatial resolution in normal subjects, which is measured clinically by best-corrected visual acuity (BCVA), which in turn provides a functional metric for foveal cones. Spectral discrimination (color vision) is another key function of cone-based vision, and it can be used to monitor cone function in retinal pathology.

The physiological basis of trichromatic color vision is the cone photoreceptor, which has three sub-types: the long- (L-), medium- (M-), and short- (S-)wavelength sensitive cones. These have spectral sensitivities with peaks at around 558 (L-cones), 531 (M-cones), and 419 nm (S-cones) (Dartnall et al., 1983). The signal from any individual cone varies in only one dimension and therefore cannot alone convey information regarding color (univariance). The visual system extracts information about the spectral quality of light by comparing the activity of the three classes of cone, a process that commences in the second-order retinal neurons, the bipolar cells (Solomon and Lennie, 2007; Simunovic, 2016). Early post-receptoral processing is proposed to occur in two molecularly, anatomically and functionally distinct subsystems of color vision (Solomon and Lennie, 2007). The first, phylogenetically ancient subsystem, compares quantal catches from the S-cones to those of the M + L-cones (tritan color discrimination). The S-cone system has poor spatial and temporal resolution, and does not normally contribute significantly to clinical measures of visual acuity (Solomon and Lennie, 2007). The second, more recent sub-system, compares quantal catches from the M- vs. L-cones (red-green color discrimination). Of note, the latter is proposed to have evolved from a mechanism specialized for extracting spatial detail from the visual scene (Mollon, 1999). Thus reductions in visual acuity caused by retinal pathology are often accompanied by acquired red-green color deficiency, whilst acquired tritan deficiency may occur in the presence of normal visual acuity (Simunovic, 2016).

Choroideremia is an X-linked chorioretinal dystrophy with a prevalence of 1 in 50,000 which is caused by mutations in the gene encoding Rab Escort Protein 1 (REP1). Patients with choroideremia have progressive centripetal loss of retinal and choroidal structure and typically have normal visual acuity until their fourth of fifth decade. REP1 is expressed in all retinal cell types, including in cones; however, choroideremia preferentially affects the rods. The differential effects of choroideremia on the rods is believed to reflect their greater dependence (compared to the cones) on normal RPE function, which is compromised early in the disease process (MacDonald et al., 2005, 2009). The rods, in turn, appear to have a role in cone survival through the secretion of trophic factors that have a protective effect on cones. The absence of these factors is thought to be the cause of secondary cone degeneration in rod-cone dystrophies, and this is exemplified by experiments demonstrating that transplantation of normal rods improves cone survival in models of inherited retinal degeneration (Mohand-Said et al., 2000). One key trophic factor has been termed “rod-derived cone viability factor” (RdCVF) (Sahel and Léveillard, 2018), a thioredoxin-like protein which is not produced by defective rods (Narayan et al., 2016). Although other mechanisms have been proposed for bystander cone degeneration, RdCVF appears to be a key player, and has been used in preclinical gene therapy studies to delay secondary cone loss (Byrne et al., 2015).

Anecdotally, choroideremia patients in the Phase I/II trials of sub-retinal gene therapy reported improvements in color saturation following treatment, suggesting that color vision may be a possible biomarker for functional rescue. Although color vision deficiency has not historically been recognized as a feature of choroideremia, it has more recently been demonstrated to result in color vision defects early in the disease course. However, this dyschromatopsia may be difficult to characterize with standard clinical tests (Jolly et al., 2015; Heon et al., 2016), both because of the demands they place on subjects, and because their results cannot be directly related to changes in the machinery of color vision.

**FIGURE 1:**
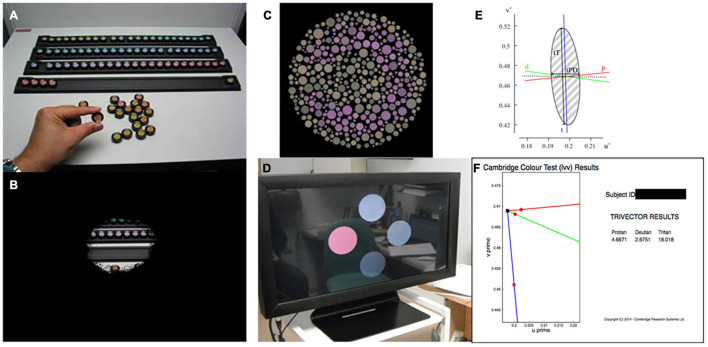
**(A)** Farnsworth-Munsell 100 Hue color vision test. **(B)** Simulation of visual field loss restricting the view of the test resulting in slow test times. **(C)** Cambridge color test (CCT) target. **(D)** Low vision version of the CCT showing four circles across the screen, providing an easier target for patients with low vision and reduced visual field. **(E)** Example output from full CCT version, producing an ellipse in color vision function in color space. **(F)** Example output from trivector test showing outputs for the protan, deutan, and tritan axes.

Color vision can be examined using a number of different tests: the choice of which depends upon the characteristics of the subject, and the likely mechanism of color vision loss (Dain, 2004). The preferred test of the US Food and Drug Administration (FDA) for the investigation of acquired color vision deficiency in clinical trials is the Farnsworth-Munsell 100 Hue (Figure 1A; Farnsworth, 1943), in part as a result of its longstanding use (Seitz et al., 2018). It consists of 85 colored caps divided across four boxes that must be arranged in order by the subject. The colored aperture of each cap subtends about 2 degrees at the recommended viewing distance, and their hues are arranged by the subject so that they appear to form a gradual progression in color. Each subject’s performance is quantified using a scoring system, which is based upon the arrangement of the caps. One drawback of the test is the large number of artefacts introduced by the unequal perceptual steps between different caps (Simunovic, 2016). Additionally, there is a high degree of inherent test variability, making reliable quantitative analysis difficult. As a consequence, large alterations are needed between visits to establish genuine change (Kinnear and Sahraie, 2002). The FM 100 Hue is particularly difficult for patients with visual field loss to perform, as the task requires a significant portion of visual search. Although many other color vision tests have been developed, not all are suitable for patients with acquired color vision deficiency (Simunovic, 2016). Fewer still are suited to patients with severely impaired visual acuity or visual field. The low vision Cambridge Color Test (lvCCT) was developed especially to assess patients with acquired color vision deficiency in whom spatial vision, or the visual field, is impaired. Loss of color discrimination in the central visual field is not directly influenced by peripheral visual field loss *per se*, though the latter presents difficulties in the conduct of many tests of color vision—as noted above, and demonstrated in Figure 1B (Swanson et al., 1993; Jolly et al., 2015).

The lvCCT is a development of the standard version of the CCT (Regan et al., 1994) (Figures 1C,D), which uses a Stilling-type array, that in turn places high(er) demands on spatial vision. The standard CCT has been used to investigate color discrimination ellipses in choroideremia, and has demonstrated evidence of preferential involvement of the tritan system of color vision (Seitz et al., 2018). The standard CCT is demanding and may not be suitable for all clinical trials participants with low vision or visual field loss (Seitz et al., 2018). Additionally, many axes (i.e., ≥8) of color space are typically probed to derive discrimination ellipses (Figure 1E), making this approach time-consuming. As an alternative, testing time may be reduced by simply assessing color discrimination along the so-called cardinal axes of color space: the tritan, deutan and protan axes (the so-called “trivector” test approach). The lvCCT (Figure 1D) has been used in clinical trials aiming to restore cone function in achromatopsia (Zein et al., 2014; Fischer et al., 2020). It can be used in patients with poor visual acuity and reduced visual fields, making it very useful for patients with retinal and visual pathway pathology (Simunovic et al., 1998; Figure 1F).

We aimed to use the lvCCT trivector test to characterize color vision loss in choroideremia, and to test its suitability as a potential outcome measure in clinical trials. Patients with, and without, significant loss of BCVA were tested: in particular, we hypothesized that patients with well-preserved visual acuity may still demonstrate tritan losses in color discrimination and that loss of red-green color discrimination may ensue once visual acuity was sub-normal (see above). Furthermore, we aimed to compare the lvCCT findings with those obtained with the currently preferred test of the US FDA, the FM 100-hue test. Finally, we also aimed to compare color discrimination to other estimates of macular function (electrodiagnostic assessment) and structure [optical coherence tomography (OCT)].

## Materials and Methods

Patients were examined as part of the screening process to assess eligibility for an ongoing retinal gene therapy trial for choroideremia (NCT02407678). Control participants were recruited from accompanying persons and staff members. Informed consent was obtained and the work adhered to the Declaration of Helsinki. All participants underwent BCVA testing with a Bailey-Lovie style visual acuity chart (Bailey and Lovie, 1976) and color vision testing with the Metropsis System (Cambridge Research Systems, Cambridge, United Kingdom). Individuals with visible media opacities, or likely congenital color vision deficiency (failure at the Ishihara test; *N* = 1) were excluded.

The control group was age-matched to the patients. Patients were divided into two groups for analysis: preserved BCVA (≥80 ETDRS letters) based on population studies defining the upper limit of normal (Brown and Yap, 1995) and reduced BCVA (<80 ETDRS letters) to explore the relationship between BCVA and acquired color vision deficiency in choroideremia (Simunovic, 2016). Examining patients with preserved BCVA permitted investigation of the earliest defects in color vision. Results for protan, deutan, and tritan axes were compared between-groups using one-way ANOVA, following log transformation. Additionally Spearman correlation of color defect and BCVA was conducted.

The full cohort consisted of 21 control participants (mean age 31 years; range 20–46 years), 35 choroideremia patients with BCVA ≥80 letters (equivalent to 0.10 logMAR, mean age 31 years; range 14–55 years), and 18 choroideremia patients with reduced BCVA (mean age 34 years; range 17–71 years). The subset of 30 patients that underwent additional testing had a mean age of 32 years (range 17–55 years), and all had a BCVA of ≥ 80 letters. Summary statistics are shown in Table 1.

**TABLE 1:** Summary characteristics of patient groups.

**Group**	**N (eyes)**	**Age (year)**	**BCVA (letters)**	**Protan threshold (×1000 CIE1976 Luv units)**	**Deutan threshold (× 1000 CIE1976 Luv units)**	**Tritan threshold (× 1000 CIE1976 Luv units)**
Control	21	31 ± 6	91 ± 4.8	3.982 ± 2.353	4.559 ± 3.151	6.725 ± 2.467
Preserved BCVA	35	31 ± 9	86 ± 3.8	13.814 ± 20.334	9.872 ± 10.447	20.813 ± 27.983
Reduced BCVA	18	34 ± 15	72 ± 9.6	17.519 ± 19.946	14.401 ± 18.283	27.602 ± 25.803
Patient subset	30	32 ± 9	83 ± 5.4	3.453 ± 7.455	3.246 ± 6.114	6.673 ± 10.808

*All data is presented as mean ± standard deviation.*

The trivector lvCCT was used to explore protan, deutan, and tritan color discrimination with the white point of the test set to the chromaticity co-ordinates of CIE D65 (*u*' = 0.209, *v*' = 0.488; CIE (1976) u' v' co-ordinates) and the confusion point set to protan (*u*' = 0.678, *v*' = 0.501), deutan (*u*' = -1.217, *v*' = 0.782) and tritan (*u*' = 0.257, *v*' = 0.0). In this test, 4 discs subtending 4 degrees at a viewing distance of 1.5 m are presented to the subject (see Figure 1D). The center of each disc is 4.3 degrees from the center of the screen. Stimuli were presented for a maximum of 20 s to allow participants adequate time to search the display, even in the presence of severe visual field loss. An auditory signal accompanied presentation of the stimulus to alert patients and to draw attention to the screen. In order to negate the possibility of subjects using perceived brightness differences to detect differences in color, the lvCCT employs luminance randomization: i.e., the four discs vary randomly in brightness. One disc—the “test disc”—was varied systematically from the remaining 3 (“background discs”) in chromaticity. The subject’s task was to determine and then indicate which disc differed from the remaining three in color *via* an attached button box. Thresholds for protan, deutan and tritan axes were established using an interleaved adaptive staircase procedure. Each staircase involved a decrease rate of 50% in hue saturation before the first reversal and a 12.5% decrease after the second reversal (fixed increase rate of 25%). Threshold was calculated as the mean of the final six, of a total of seven, reversals. If the subject was unable to correctly determine the position of the most saturated test disc on five consecutive occasions, testing along that axis was terminated and the threshold set at the maximum saturation. Participants undertook a training run before performing the actual test to familiarize themselves with the task.

A subset of patients underwent both the FM 100 Hue and lvCCT. The FM 100 Hue (DG Color, Wiltshire, United Kingdom) was set at a viewing distance of 50 cm and took place within a lighting cabinet approximating CIE standard illuminant D. Caps were randomized by the examiner prior to testing, and placed back in the tray. The participant was instructed to arrange the caps in a gradual progression in color between the fixed end caps. The order of the caps was recorded using the accompanying custom software to calculate the total error score. The tests were subsequently classified as normal or abnormal based on published values (Kinnear and Sahraie, 2002), and calculated values for the upper 95% confidence limit of normal from the normative data collected above. Normative data for the lvCCT test has been reported by Paramei and Oakley (2014). Additionally, the reported variation across the age ranges represented by our cohort is small. A kappa agreement between tests was calculated.

The same patient subset also underwent ISCEV (International Society for the Clinical Electrophysiology of Vision) pattern electroretinogram (PERG) testing (Bach et al., 2013) with both standard (15 degrees × 12 degrees) and large field (30 degrees × 24 degrees) checkerboard stimuli (Lenassi et al., 2012), using the P50 component as an objective measure of suprathreshold cone-driven macular function and N95 as a measure of retinal ganglion cell function. Recordings were obtained using gold foil recording electrodes using a constant check size of 0.8 degrees. Testing was performed using the Espion system (Diagnosys LLC, Lowell, MA). The key features of the PERG are P50 peak time and amplitude as markers of macular dysfunction. Additionally, N95 is indicative of ganglion cell dysfunction.

The color vision results for each axis were treated as Cartesian co-ordinates in CIE (1976) u', v', and a combined vector calculated with | | CV| | = √(((protan^2^ + deutan^2^)/2) + tritan^2^), where | | CV| | is the absolute color vision vector length. Protan and deutan results were averaged to reduce the impact of weighting toward L/M defects. A Spearman regression model examined the relationship between the PERG and the color vision vector combining all axes.

**FIGURE 2:**
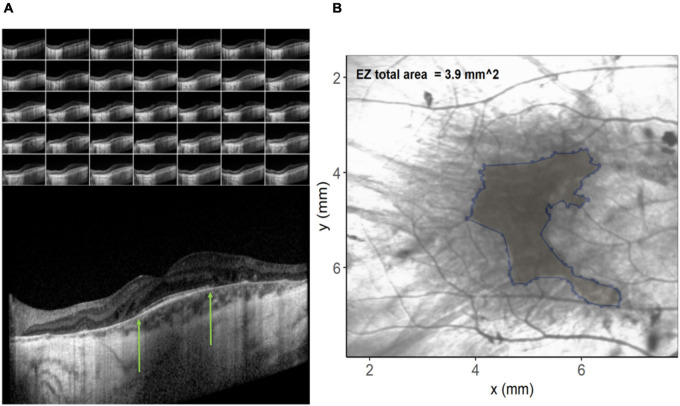
**(A)** Images on the left show the individual b-scan slices from an optical coherence tomography (OCT) volume with the central slice expanded. The green arrows denote the edges of the ellipsoid zone. These co-ordinates from each of the slices are plotted on the infra-red image on the right **(B)** and connected to create an ellipsoid area across the OCT volume.

In order to investigate possible structural correlations to color discrimination, a custom program was written in the R programming language (v3.6.3) (R.C. Team, 2017). Spectral domain optical coherence tomography (SD-OCT) images were acquired during screening using the Spectralis OCT volume (Heidelberg Engineering, Heidelberg, Germany). Data were exported as XML files and each OCT b-scan per patient (Figure 2A) manually inspected to identify coordinates of the limiting edges of the intact ellipsoid zone (EZ). These coordinates were input into the custom program, resulting in a polygon overlaid on the en-face image (Figure 2B) representing the area of the ellipsoid zone across all B-scans in a volume. In difficult cases where the ellipsoid zone termination was unclear, the profile method detailed by Smith et al. (2019) was employed. The relationship between EZ and color vision was examined using Spearman’s rank-order correlation coefficients. EZ area was utilized to order avoid the confounding effects of retinal remodeling on thickness measurements (Jolly et al., 2020). EZ is a measure of remaining retina.

Groups were compared using a two way ANOVA. Due to unequal variances, *post hoc* analysis was conducted with Games-Howell comparisons. All results are reported for the right eye only. All statistical analyses were conducted in SPSS (version 25.0, IBM Software, New York, NY, United States) or GraphPad Prism 9 (GraphPad Software, San Diego, CA, United States) unless stated otherwise.

## Results

### Comparison Between Groups Shows Early Tritan Loss

**FIGURE 3:**
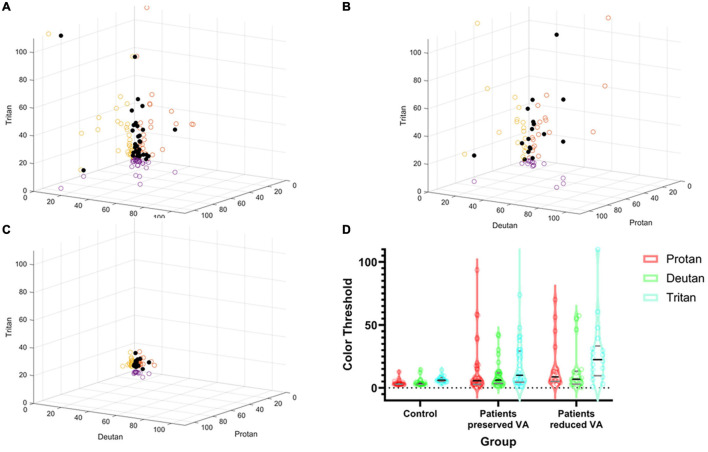
Summary of color discrimination results. Spread of individual data points plotted against protan, deutan, and tritan scores on the X, Y, and Z axes, respectively (black dots). The circles on the axes walls show the corresponding shadows of the results on each axes separately. **(A)** Choroideremia eyes with preserved BCVA. **(B)** Choroideremia eyes with reduced BCVA. **(C)** Control eyes. The higher the number, the greater the degree of the color defect against the corresponding axis. **(D)** Pirate plot of group differences. The outer envelope shows the spread of the raw data with the widest point showing the mean and the black line representing the median. The gray lines represent the interquartile range. Additionally, dots represent each raw data measurement point.

**FIGURE 4:**
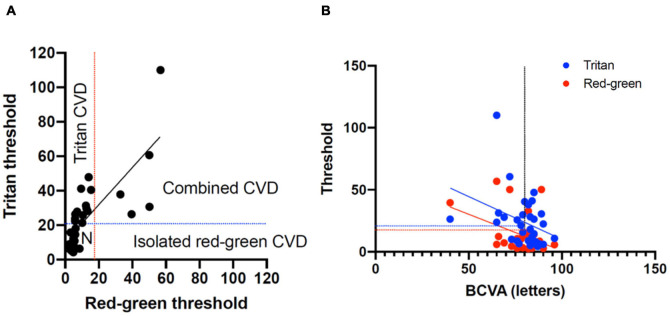
**(A)** Classification of color vision defects (CVD) measured with the low vision Cambridge Color Test (lvCCT) with the regions of normal color vision, tritan loss, red-green loss, and combined loss labeled. **(B)** Correlation of best-corrected visual acuity (BCVA) with red-green and tritan color defects. The vertical dotted line shows 20/20 visual acuity. The blue and red lines show the upper 95% cutoff of normal for the tritan and red-green loss, respectively.

Figure 3 shows the spread of the raw lvCCT color discrimination data, with the highest threshold along the tritan axis in all cohorts as shown by the longest vector lines aligned with the vertical axis. There was greater variation in color discrimination thresholds in the choroideremia patients than the normal controls. Group averages are shown in Figure 3B. There was a statistically significant difference between groups, as determined by two-way ANOVA for protan [*F*(2, 53) = 9.09, *P* < 0.01], deutan [*F*(2, 53) = 6.44, *P* < 0.01], and tritan [*F*(2, 53) = 19.54, *P* < 0.01] thresholds. A Games-Howell *post hoc* test revealed that the color discrimination was significantly worse in both the “preserved” and reduced BCVA cohorts compared to the control group for all three color confusion axes (*P* ≤ 0.05). However, the two choroideremia cohorts were not significantly different for any of the axes (*P* > 0.05). Figure 4A demonstrates the classification of the color defect for each eye with choroideremia and shows that a higher proportion had a tritan defect compared to classification of a red-green defect.

The Spearman correlation co-efficient for BCVA and combined red-green vector length and tritan vector length was -0.59 (-1.1 to -0.12 95% confidence interval) and -0.46 (-0.92 to 0.001 95% confidence interval), respectively. This was significantly for red-green (*P* = 0.02) but not for tritan (*P* = 0.05), consistent with the change in a color defect independent to BCVA (Figure 4B).

### Comparison With 100 Hue Reveals the Cambridge Color Test Is More Sensitive

**FIGURE 5:**
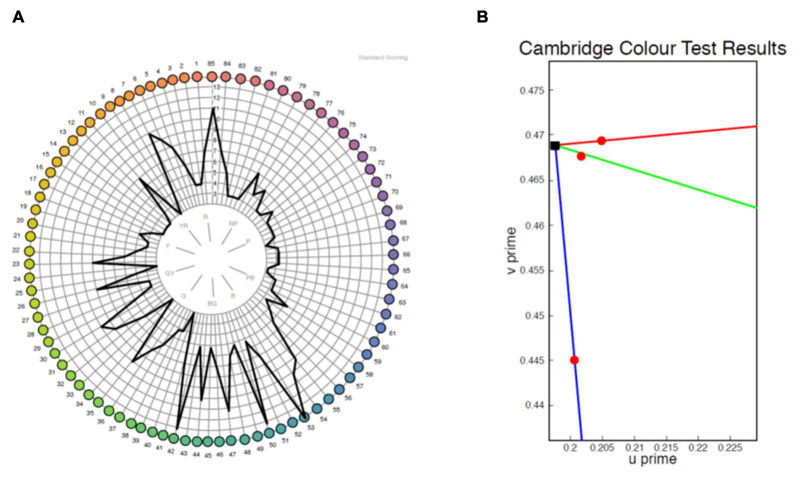
Representative sample of a test result for the same eye from the FM 100 Hue **(A)** and low vision Cambridge Color Test (lvCCT) **(B)**. The FM 100 Hue does not have a clearly definable axis of color loss, whereas the lvCCT results indicate a reduced tritan score (longer vector) but normal protan and deutan scores as shown by the short vectors.

The majority of patients demonstrated abnormal results on both tests (Table 2). A clear axis of defect on the FM 100 Hue was not evident in any of the eyes (representative test result for the 100 Hue and lvCCT for the same eye shown in Figure 5). Out of the 19 eyes classified as abnormal by the lvCCT, 26% were classified as having a clear tritan defect, 11% as having a protan defect, 16% as having a deutan defect and 47% as having a defect along two or more axes. In all instances of the latter, tritan discrimination was affected. The kappa agreement coefficient, *k* = 0.3878 (*P* = 0.03) indicated only fair agreement between the FM 100 Hue and CCT.

**TABLE 2:** Classification of eyes as normal or abnormal when tested with the 100 Hue and Cambridge Color Test (CCT).

	**lvCCT abnormal**	**lvCCT normal**
100 Hue abnormal	17	6
100 Hue normal	2	5

### No Correlation Between Color Vision and Pattern Electroretinogram

In order to examine suprathreshold macular function objectively, PERG was conducted on the subset that went additional testing and had preserved BCVA eyes. In response to the standard field PERG stimulus, the P50 and N95 were measurable in 26 out of 30 eyes, with the remaining 4 showing a flatline response. Mean P50 peak time for the cohort was 56.4 ± 12.7 ms with a mean P50 amplitude of 0.77 ± 0.49 μV. The mean amplitudes were subnormal based on local reference ranges (x-x ms and y-y μV, respectively) and the Espion normative database (Lenassi et al., 2012). The N95 amplitude for the cohort was 1.89 ± 1.17 μm, suggesting a preserved N95:P50 ratio, indicating preserved retinal ganglion cell function. The multiple regression model demonstrated no significant relationship between any of the PERG parameters with | | CV| | (*R* = 0.16, *P* = 0.90). Coefficients were -0.14 [-7.4 to 7.1], *P* = 0.96 for P50 peak time, 43.0 [-284.4 to 198.4], *P* = 0.72 for P50 amplitude, and 32.0 [-65.8 to 129.8], *P* = 0.50 for N95 amplitude.

### Optical Coherence Tomography Ellipsoid Zone Area Does Not Correlate to Color Discrimination

**FIGURE 6:**
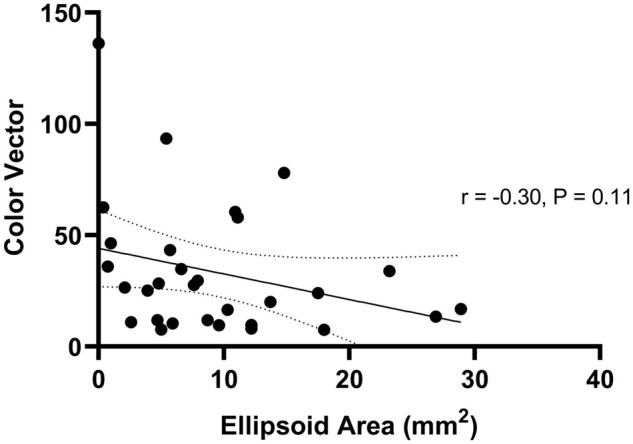
Regression relationship between color vector and ellipsoid area in choroideremia. The color vector represents the degree of color vision loss across the three axes with a higher number representing a greater degree of color loss. The solid line is the regression line and 95% confidence intervals are shown with the dotted lines.

Thirty patients with choroideremia had OCT images from the same day as color vision testing with sufficient quality to enable qualitative delineation of the retinal layers. Measurement of the ellipsoid area was performed using the process described in the methods section. Images on which the ellipsoid zone could not be reliably commented on were excluded from imaging analysis. The color vector was plotted against the ellipsoid area (Figure 6) and showed a negative trend. The slope of the regression line indicated a reduction in color vector of -1.15 for each unit reduction in ellipsoid area (95% confidence interval -2.59 to 0.29); the relationship was not statistically significant (*P* = 0.11). The findings remained non-significant when each color axis was measured separately.

## Discussion

This study uses color discrimination, as assessed by the lvCCT, to characterize dyschromatopsia in 53 choroideremia patients, with and without, significant loss of visual acuity. The results were compared to those obtained using more established methods, including the Farnsworth Munsell 100-Hue test and the ISCEV-standard pattern ERG measure of macular function. The lvCCT results were also compared with a spectral-domain OCT measure of macular preservation, with an overall view to assess the potential suitability of color discrimination as an outcome measure in clinical trials.

The lvCCT rapidly characterized color discrimination (mean testing time 5 min) along the cardinal axes of color space. By contrast, the FM 100 Hue took longer to perform (over 30 min) and provided results that could not directly be related to losses of discrimination in either of the two subsystems of color vision (M vs. L-cones, measured *via* protan and deutan axes, and S-cone vs. M + L-cone, assessed *via* the tritan axis). Furthermore, the two tests only demonstrated fair agreement.

The majority patients had tritan color defects, which is in keeping with previous reports in rod-cone dystrophies (Swanson et al., 1993; Seitz et al., 2018). The vectors in the S cone direction could be compared to the L and M cone directions due to the use of a normal control group, which allows the use of the color vector | | CV| | to be used as a valid approach. All color spaces include the assumption that just noticeable differences are additive. There are several possibilities that should be considered to account for this finding. Although cataract is a feature of some retinal dystrophies, none of the included patients had significant media opacity: hence absorption by lens pigment cannot account for our observation. It has been previously noted that the S-cones appear to be especially vulnerable to retinal pathology, and there are several hypotheses which have attempted to account for this observation (Mollon, 1982; Simunovic, 2016). Part of the apparent vulnerability of S-cone mediated visual function to pathology appears to arise from adaptational differences between the S-cone mechanism and the M-/L-cone mechanism and biases introduced by testing approaches. Mollon hypothesized that certain tests (e.g., those employing yellow backgrounds) may place the S-cone mechanism at disadvantage through adaptation of post-receptoral mechanisms to one extreme of their operational range. Such a hypothesis cannot be invoked to explain our data, however, as neutral adaptation conditions were used. However, adaptation asymmetries have also been identified for neutral/white backgrounds (Kalloniatis and Harwerth, 1989; Simunovic, 2016; Simunovic et al., 2021). In particular, the S-cone mechanism is more likely to be probed under conditions where it does not display Weber-like adaptation. This may lead to apparent selective pathology through adaptational asymmetry (Kalloniatis and Harwerth, 1989; Simunovic, 2016; Simunovic et al., 2021); this asymmetry may account for at least part of the apparent vulnerability of the S-cones. Another hypothesis—the so-called “vulnerable photoreceptor” hypothesis—supposes that the S-cones are inherently more susceptible to pathological processes. For example, S-cones have been reported to be more susceptible to metabolic damage (Hood and Greenstein, 1988). Furthermore, it has been suggested that S-cones may be preferentially affected by rod-selective pathology. Part of this shared “vulnerability” may be driven by physiological similarities between the rods and the S-cones; for example, S-cones and rods share similarities in distribution which may render them similarly vulnerable to topographically variable pathological processes (Curcio et al., 1991; Hofer et al., 2005). Furthermore, S-cones and rods share similarities in the expression of certain proteins, e.g., carbonic anhydrase (Nork et al., 1990), which may place them under similar pathophysiological stress under certain conditions. Furthermore, both S-cones and rods derive from a common precursor cell, as evidenced by patients with Nr2E3 mutations, who have an increased S-cone complement, but a grossly abnormal rod complement (Sohn et al., 2010). There is an increase in the inflammatory response following rod death which has a greater effect on S-cones compared to L- and M-cones (Di Pierdomenico et al., 2020). Cone cells have access to an alternative Mũller cell-mediated visual cycle, which reduces their reliance on the RPE for chromophore recycling. Mũller cells have a lower association with S-cones compared to other cone types, which may make them more vulnerable RPE degeneration (Lindenau et al., 2019) in choroideremia. In view of the differences between S-cones and other cone types, and simultaneous similarities with rods, it is possible that S-cones may be preferentially affected by primary rod disease, either *via* inflammatory processes or other degenerative processes.

Dyschromatopsia was evident prior to reductions in BCVA in many choroideremia subjects; for this reason, it may prove to be a suitable functional biomarker for very early abnormalities in macular function. In choroideremia, foveal architecture is observed to be disrupted before BCVA loss occurs (typically retinal thickening with blunting of the foveal depression). This in turn suggests that other aspects of visual function may better reflect early macular pathology in choroideremia and therefore highlights the importance of this work (Jacobson et al., 2006). In particular, it has previously been noted that tritan color vision deficiency typically occurs in patients with well-preserved BCVA. It has been argued that this reflects in part the underlying physiology of color processing. In particular, the S-cones comprise only a small proportion of the cone complement (about 4%) and their loss will not affect spatial resolution (BCVA) in the majority of patients. However, the M-/L-cone mechanism has been proposed to be parasitic upon a pathway which initially evolved to extract spatial detail (Mollon, 1999). For this reason, loss of M-/L-cone mediated color discrimination is often seen in patients who have concurrent loss of BCVA (Marré and Pinckers, 1985; Simunovic, 2016).

All patients with choroideremia had an abnormal pattern ERG P50 component, consistent with macular dysfunction, including those with preserved BCVA. However, the severity of the PERG reduction does not correlate with color discrimination at the lvCCT; this is in keeping with previous findings (Falcao-Reis et al., 1991). PERG and psychophysical measures of macular function have been reported to be discordant, and this may reflect the different areas assessed by the two modes of testing (Ciavarella et al., 1997). We have previously demonstrated a relationship between color vision defect with central microperimetry threshold (Jolly et al., 2015). Microperimetry is likely reflective of cone function in the area tested so is probably exploring a more comparable cone population (Simunovic et al., 2016; Han et al., 2018). Different functional measures test different aspects of vision over different regions of the visual field so will not necessarily show good correlation. Additionally, previous work has shown autofluorescence area of preservation and location in relation to the fovea appeared to play a role in explaining color vision losses in choroideremia. This may reflect the fact that more advanced FAF changes correlate to decreased photoreceptor function/visual pigment cycling (Jolly et al., 2015).

The OCT ellipsoid area was utilized as an indirect marker of cone presence, and the direction and extent of association was examined in relation to color discrimination. Although a negative correlation was shown, this was not statistically significant. Ellipsoid area in part reflects the disease stage, it may correlate poorly to color discrimination for two reasons. First, if such changes occur outside of the retinal areas probed by the lvCCT, no correlation would be anticipated. Second, early functional alterations in the cones may be indicated by changes other than complete obliteration of the ellipsoid layer (e.g., fundus autofluorescence).

In summary, tritan defects are seen early in choroideremia, prior to BCVA loss. This finding is generally in keeping with our current understanding of the machinery of color vision, the known adaptational asymmetries in the S- and M-/L-cone subsystems of color vision and the dependence of cones on the rods for survival. We note that several patients who have undergone sub-retinal *AAV2.REP1* gene “replacement” therapy have reported subjective improvements in color saturation following surgery, which has prompted us to explore color discrimination as part of an ongoing Phase 2 trial. Color vision appears to be an early biomarker of macular dysfunction in choroideremia and may be more sensitive than BCVA when used for monitoring gene therapy outcomes. We note that other approaches could be used to explore these subjective reports in color vision. For example, patients with symmetrical vision loss could undergo testing to assess inter-ocular differences in perceived saturation following first-eye surgery. Where this is not possible, exploration of white-points/long term color constancy could be explored. Furthermore, changes in receptoral function could be determined by assessing threshold-vs. intensity functions pre-, and post-surgery, to elucidate changes in the S-, M-, and L-cones as well as the rods. Such testing may also help to identify the site of losses of sensitivity (i.e., d_1/2_, or receptoral vs. d_3_, or post-receptoral), as previously outlined (Seiple et al., 1993; Simunovic et al., 2021).

In summary, this study quantifies color vision deficits in patients with choroideremia with both preserved and reduced visual acuity, and reveals a high incidence of S-cone/tritan pathway dysfunction. The lvCCT trivector test provides a rapid means of quantifying color discrimination along the cardinal axes of color space in this patient group. Furthermore, it identifies losses in central macular function in patients prior to the loss of visual acuity or evidence of structural change on OCT. This highlights the potential use of color discrimination as an outcome measure in future clinical trials which target sub-retinal gene therapy to the macula.

## Data Availability Statement

The raw data supporting the conclusions of this article will be made available by the authors, without undue reservation.

## Ethics Statement

The studies involving human participants were reviewed and approved by the Health Research Authority 15/LO/1379. The patients/participants provided their written informed consent to participate in this study.

## Author Contributions

JJ designed the study, collected and analyzed the data, and wrote the manuscript. AR and AJ contributed to the data collection. AJ, MS, and AD contributed to the data analysis. AR, AD, HB, and RM provided input on the test procedures and analysis techniques. MS contributed to the writing of the manuscript. All authors reviewed the manuscript.

## Author Disclaimer

The views expressed are those of the authors and not necessarily those of the NHS, the NIHR or the Department of Health. The sponsor and funding organization had no role in the design or conduct of this research.

## Conflict of Interest

RM is a consultant in retinal gene therapy to Novartis, Gyroscope Therapeutics, and Biogen, but these companies had no input into the work presented. The remaining authors declare that the research was conducted in the absence of any commercial or financial relationships that could be construed as a potential conflict of interest.

## Publisher’s Note

All claims expressed in this article are solely those of the authors and do not necessarily represent those of their affiliated organizations, or those of the publisher, the editors and the reviewers. Any product that may be evaluated in this article, or claim that may be made by its manufacturer, is not guaranteed or endorsed by the publisher.
